# Supplementary material for: The Symbiotic Bacteria—Xenorhabdus nematophila All and Photorhabdus luminescens H06 Strongly Affected the Phenoloxidase Activation of Nipa Palm Hispid, Octodonta nipae (Coleoptera: Chrysomelidae) Larvae
Source: Pathogens. 2023 Mar 23;12(4):506. doi: 10.3390/pathogens12040506 (PMC10142170; doi:10.3390/pathogens12040506)
Supplement: Supplementary file 1 [file pathogens-12-00506-s001.zip › pathogens-2210384-supplementary.pdf]

Table S1. Recognition gene and ProPhenoloxidase activation genes primers

Gene Name	Forward Primer (5’→3’)	Reverse Primer (5’→3’)
**qCTL**	TGGACTAAACCCCGGTCTTC	ACAGGGCTAGGGAAAACTCC
**qSPP56**	CGGTTGGTGGAAAGTGTCAG	CCCTCGTTGTCCAGCTTCTA
**qSPI28**	TCGCCTTAGTGATAGCGTGT	ACAGGGCTAGGGAAAACTCC
**qPPAF1**	GATCACCGGCGACAAAGAAA	CAGCTTGTTGGGATTGCCTT
**qPPO**	GTATCTTGTCACCCAATAGAGC	AAACGATTCAAGATGCCTGT
**q-RPS3-F**	GACGGTGTCTTCAAAGCTGA	ATTTCTGTACGTGTCGGGGT
